# Transforming Niclosamide through Nanotechnology: A Promising Approach for Long COVID Management

**DOI:** 10.1002/smll.202410345

**Published:** 2025-05-19

**Authors:** Sanoj Rejinold N, Goeun Choi, Geun‐woo Jin, Jin‐Ho Choy

**Affiliations:** ^1^ Intelligent Nanohybrid Materials Laboratory (INML) Department of Chemistry School of Science and Technology Dankook University Cheonan 31116 Republic of Korea; ^2^ R&D Center Hyundai Bioscience Co. LTD. Seoul 07990 Republic of Korea; ^3^ Division of Natural Sciences The National Academy of Sciences Seoul 06579 Republic of Korea

**Keywords:** COVID‐19, broad‐spectrum antiviral therapy, global impact, long COVID, nanoengineered niclosamide, post‐COVID‐19 syndrome

## Abstract

Coronavirus disease 2019 (COVID‐19), caused by severe acute respiratory syndrome coronavirus 2 (SARS‐CoV‐2), has infected over 770 million people worldwide. The long‐term effects of COVID‐19 and their management have become important issues. Accumulating evidence indicates that post‐COVID‐19 syndrome, also known as long COVID, is not limited to respiratory symptoms but affects a wide range of systems, including neurological, cardiovascular, gastrointestinal, musculoskeletal, and reproductive systems etc. The social and economic losses associated with these effects are estimated to reach 3·7 trillion dollars in the United States alone. However, no treatment for long COVID has been developed. Herein, the literature on long COVID is comprehensively reviewed to examine the underlying causes. Additionally, evidence supporting the efficacy of nanoengineered niclosamide is presented, given its ability to counteract the underlying causes. Niclosamide is already Food and Drug Administration (FDA)‐approved, and the nanoengineered one is a viable candidate for clinical trials for long COVID.

## Graphical abstract

This review highlights the transformation of niclosamide, a repurposed drug, through molecular science strategies to overcome its pharmacokinetic limitations. Emphasizing novel nanohybrid technologies and delivery platforms, the article explores their therapeutic potential in targeting the complex pathophysiology of long COVID, paving the way for effective antiviral and anti‐inflammatory interventions.

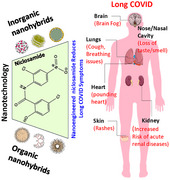

## 1 Introduction

Long COVID, a chronic condition that persists after acute SARS‐CoV‐2 infection, is characterized by prolonged symptoms affecting multiple organ systems. It is distinct from COVID‐19 (Coronavirus Disease 2019), which refers to the acute phase of the infection caused by the SARS‐CoV‐2 virus. Long COVID persists for at least 3 months after SARS‐CoV‐2 infection.^[^ ^1^ ^]^ The symptoms of long COVID are highly diverse, with over 200 symptoms identified, including brain fog, post‐exertional malaise, and manifestations affecting the respiratory, neurological, cardiovascular, gastrointestinal, musculoskeletal, and reproductive systems,^[^ ^2^, ^3^ ^]^ (**Table** **1**) caused by persistent viral presence, excessive inflammation, and immune system modulation, etc via SARS‐CoV‐2.^[^ ^4^ ^]^ It has been, therefore, estimated that the societal cost of long COVID was $3·7 trillion based on various factors, such as quality of life, lost earnings, and medical care spending, corresponding to 17% of the 2019 gross domestic product in the United States, according to the report released earlier in 2022.^[^ ^5^ ^]^ Despite these significant social and economic losses, no effective treatments for long COVID have yet been identified till now. Though there are various medications available to alleviate long COVID, the focus has been made on niclosamide, a drug that has been shown to reduce the viral load, inflammatory responses, and immune system disruption caused by the SARS‐CoV‐2, which are the underlying causes of the various long COVID symptoms.

**Table 1:** Plausible therapeutic mechanisms of nanohybridized niclosamide (NIC) in addressing the wide range of symptoms associated with long COVID. This table maps the diverse and multisystemic symptoms of long COVID—including systemic, pulmonary, neurological, gastrointestinal, vascular, and musculoskeletal manifestations—to the potential pharmacological activities of NIC. These include antiviral activity (reducing viral load),^[^ ^7^, ^8^ ^]^ autophagy regulation (clearing damaged cells and viral remnants),^[^ ^9^, ^14^ ^]^ mitochondrial modulation (restoring cellular energy metabolism),^[^ ^12^, ^13^ ^]^ and anti‐inflammatory and immunoregulatory effects (via STAT3 inhibition and T cell modulation).^[^ ^10^, ^11^, ^15^, ^16^ ^]^ NIC's neuroprotective actions include the ability to cross the blood‐brain barrier and reduce neuroinflammation by inhibiting microglial activation and promoting dopaminergic neurite growth.^[^ ^18^, ^19^ ^]^ Furthermore, NIC exhibits anticoagulant properties by preventing spike‐induced platelet activation via TMEM16F inhibition.^[^ ^19^ ^]^ Collectively, these mechanisms position NIC as a promising candidate to target the complex pathophysiology of long COVID, supported by referenced mechanistic studies.

		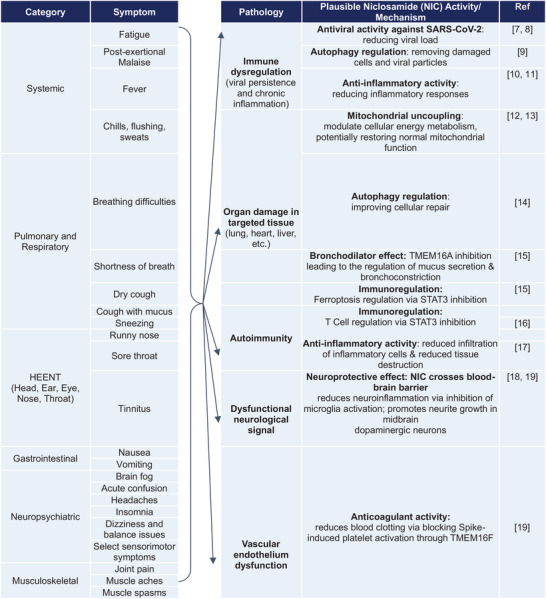			

According to the drug repurposing strategy by Jeon et al., niclosamide was found to be most effective against SARS‐CoV‐2 out of 24 potential anti‐viral drug candidates (FDA approved)^[^ ^6^ ^]^. Such a strategy could also be highly advantageous in reducing R&D time and costs, expediting availability. Niclosamide was originally a medication used to treat parasitic infections and therefore has a known safety profile.^[^ ^20^, ^21^ ^]^

However, the longstanding 60‐year challenge of niclosamide's poor solubility has significantly limited its broader therapeutic potential^7^. To address this issue, nanoengineering strategies have been developed, allowing niclosamide to be engineered at^[^ the molecular level to enhance its solubility. This approach not only improves its bioavailability but also amplifies its overall pharmacological effects, offering a more effective solution for its clinical application. Thus, nanoengineered niclosamide could help in mitigating the significant social and economic losses associated with long COVID.

**Figure 1:**
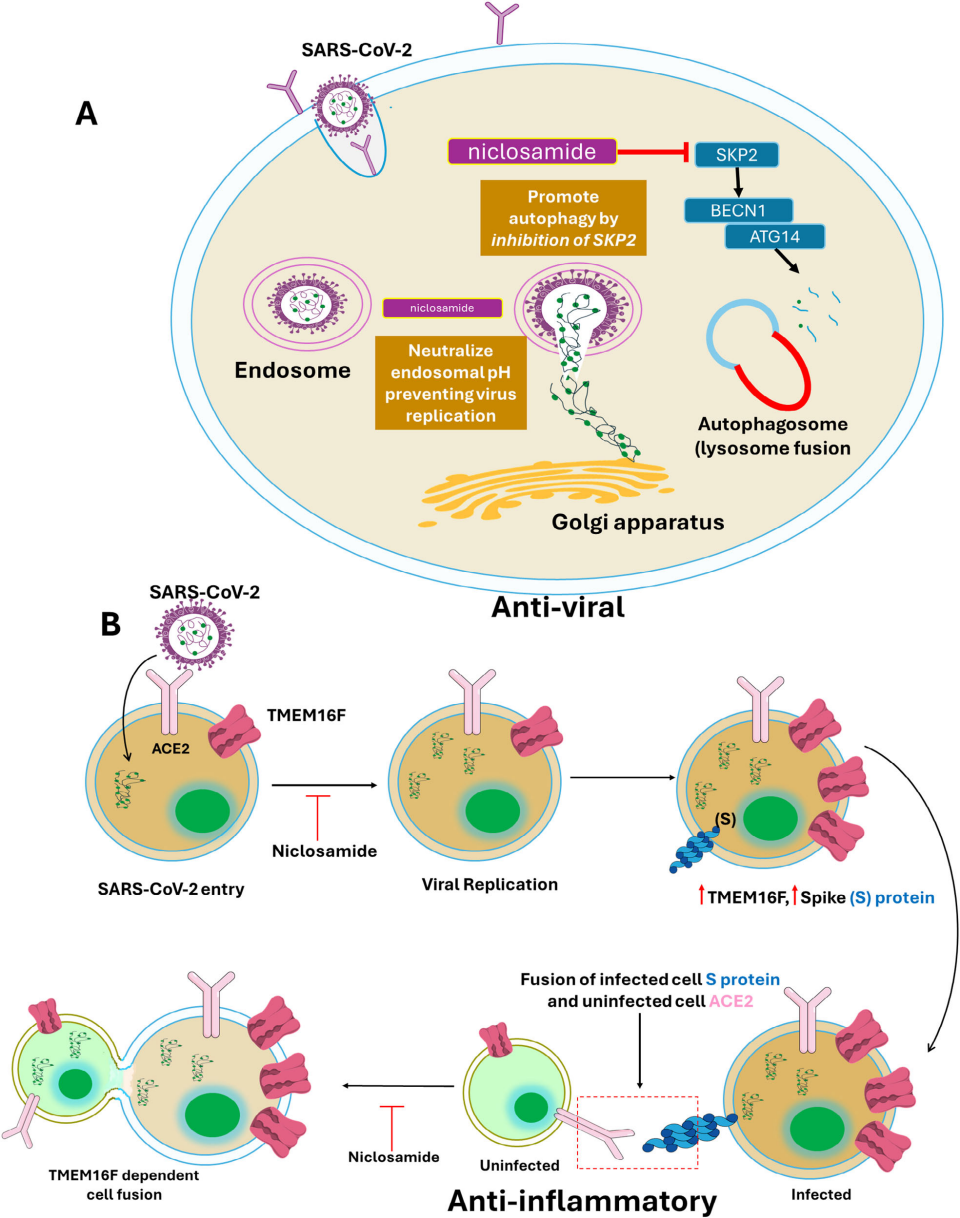
Potential mechanisms underlying antiviral and anti‐inflammatory activities of niclosamide. These include A) neutralizing endosomal pH to block viral replication, promoting autophagy by inhibiting S‐phase kinase‐associated protein 2 (SKP2), B) Diagram of niclosamide effect on SARS‐CoV‐2 entry and spike protein‐mediated syncytia formation.

Given these advantages, it is essential to actively investigate the efficacy of niclosamide as a therapeutic option for long COVID. Herein, we review the diverse factors involved in long COVID and propose nanoengineered niclosamide as a highly promising treatment. The core premise of this review is that while niclosamide in its pristine form has shown promise, its clinical translation has been hindered by poor bioavailability and systemic stability. This limitation is what nanoengineered niclosamide aims to overcome, making it a potentially viable therapeutic strategy for long COVID. **Table** **2**. shows the distinction between the established antiviral properties of niclosamide and (**Figure** **1**) the proposed benefits of its nanoformulation.

**Table 2:** Highlighting the distinction between the established antiviral properties of niclosamide and the proposed benefits of its nanoformulation.

Aspect	Established antiviral properties of niclosamide	Refs.	Proposed (or early reports) /Benefits of nanoengineered niclosamide	Refs.
Mechanism of Action	Inhibits viral replication by disrupting endosomal acidification and autophagy.	[23]	Enhanced cellular uptake and sustained drug release improve antiviral action.	[24]
Pharmacokinetics	Poor bioavailability due to low aqueous solubility and rapid metabolism.	[25]	Increased solubility and extended circulation time enhance drug exposure.	[26]
Therapeutic Efficacy	Effective in vitro against various viruses, but limited success in clinical settings due to delivery challenges.	[27]	Targeted delivery improves drug concentration at the infection site, boosting efficacy.	[28]
Toxicity Profile	Well‐established safety but potential gastrointestinal side effects at high doses.	[29]	Controlled release and reduced systemic exposure minimize adverse effects.	[30]
Administration Challenges	Requires high oral doses, leading to inconsistent plasma levels.	[31]	Nano formulations (liposomes, micelles, nanogels) enable lower doses with improved stability and absorption.	[32]

## 2 What is Long COVID?

**Scheme 1:**
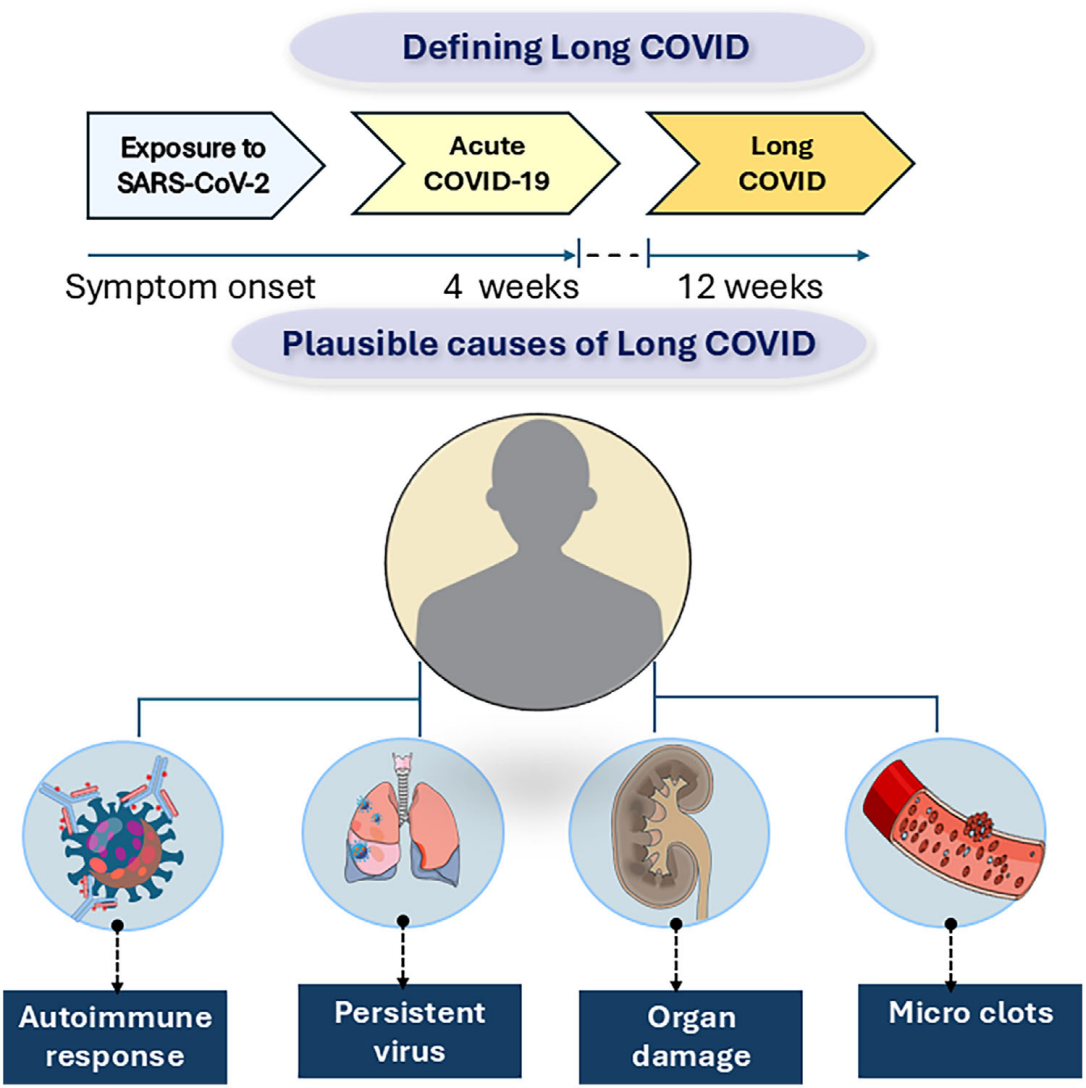
Defining long COVID A‐ and its plausible causes.

The term long COVID refers to the persistence of diverse symptoms (Table 1, **Scheme** **1**) that often appear weeks or months after the acute phase of SARS‐CoV‐2 infection.^[^ ^1^, ^32^, ^33^, ^34^, ^35^, ^36^, ^37^, ^38^, ^39^, ^40^, ^41^, ^42^ ^]^ Although some respiratory or gastrointestinal illnesses share similar symptoms to those of COVID‐19, the recovery process is marked by the emergence of new symptoms related to the neurological, cardiovascular, and reproductive systems. Various definitions for long COVID have been suggested. The World Health Organization (WHO) defines it as the persistence of symptoms or the development of new symptoms three months after the initial SARS‐CoV‐2 infection, with these symptoms lasting for at least two months. In the UK, the National Institute for Health and Care Excellence (NICE), the Scottish Intercollegiate Guidelines Network (SIGN), and the Royal College of General Practitioners (RCGP) define the first four weeks after a SARS‐CoV‐2 infection as acute COVID‐19, the period from 5 to 12 weeks as “ongoing symptomatic COVID‐19,” and symptoms lasting beyond 12 weeks as “post‐COVID‐19.”

Although many patients experience long COVID, its diagnosis is challenging. In a study in the United States, almost 33% of patients reported persistent symptoms during a 60‐day follow‐up after discharge.^[^ ^43^ ^]^ In France, a 60‐day follow‐up study showed that 66% of individuals recovering from non‐severe COVID‐19 reported persistent symptoms.^[^ ^44^ ^]^ Most patients show negative PCR test results; therefore, the diagnosis of long COVID cannot be confirmed by the PCR status. The diagnosis and assessment of long COVID are challenging owing to the wide range of over 200 symptoms and the heterogeneity among individuals.^[^ ^45^ ^]^ The wide variety of symptoms makes the establishment of a standardized diagnostic test difficult. Diagnosis is typically performed through the inclusion and exclusion of symptoms; thus, specific symptoms must be ruled out prior to confirming long COVID as a diagnosis.^[^ ^46^ ^]^

## 3 Causes Of Long COVID

### 3.1 Long‐Term Persistence of Sars‐Cov‐2

Viruses often persist within a host for months or even years. However, acute viruses lack mechanisms for persistence over extended periods within the host and thus are typically cleared from the host. Nonetheless, in some cases, acute viruses can remain in the body for extended periods. This persistence can be asymptomatic or associated with late progressive disease.^[^ ^47^, ^48^ ^]^

Similarly, studies have shown that SARS‐CoV‐2 can persist within the host for extended periods. According to Swank et al., ≈60% of patients with long COVID symptoms have detectable fragments of the SARS‐CoV‐2 virus in their blood up to 12 months post‐infection.^[^ ^49^ ^]^ Additionally, researchers from Stanford Medical School found viral RNA in the stool samples of 83% of patients with COVID‐19, with ≈4% of participants still showing viral RNA in their stool up to 7 months after diagnosis.^[^ ^50^ ^]^ The detection of viral protein fragments or RNA long after infec‐15–15‐tion suggests that SARS‐CoV‐2 may remain in the body for a considerable amount of time. Proa et al. pointed out that if there is a “reservoir” in the body, the replicating virus and/or viral RNA can produce viral proteins, which can be released into circulation.^[^ ^51^ ^]^

### 3.2 Dysregulated Immune Response

Another cause of long COVID is immune modulation and inflammation resulting from SARS‐CoV‐2 infection.^[^ ^52^, ^53^ ^]^ Fernández‐Castañeda et al. reported elevated levels of the inflammatory protein CCL11 in the blood of COVID‐19 survivors experiencing brain fog.^[^ ^54^ ^]^

Additionally, Monje et al. evaluated mice infected with COVID‐19, nine patients who died from COVID‐19, and 48 patients with brain fog symptoms due to COVID‐19; they found signs of brain inflammation in all three groups, suggesting that brain fog, a symptom of long COVID, is caused by excessive inflammation.^[^ ^55^ ^]^ Supporting the idea that brain fog is caused by excessive inflammation, Lee et al. obtained similar findings in a study of brain tissue from deceased patients with COVID‐19, revealing inflammation due to the activation of immune substances (complement) and subsequent damage to brain blood vessels.^[^ ^56^ ^]^

Wang et al. found that patients with COVID‐19 have higher levels of autoantibodies than those in uninfected individuals. Unlike antibodies that attack invading bacteria, autoantibodies are abnormal antibodies that attack the body's own organs and tissues.^[^ ^57^ ^]^ These results suggest that SARS‐CoV‐2 can affect the immune system and cause inflammation, potentially impacting the entire body, including the brain.

## 4 Candidate Treatments for Long‐Covid Undergoing Clinical Trials

As shown in **Table** **3**, various candidate treatments are currently undergoing clinical trials. These candidate drugs exhibit diverse mechanisms of action, reflecting the complexity and multifaceted nature of long COVID. Most candidates aim to address either the long‐term persistence of SARS‐CoV‐2 or inflammation, which are the primary causes of long COVID. Antiviral agents like nirmatrelvir/ritonavir,^[^ ^58^ ^]^ remdesivir,^[^ ^59^, ^60^, ^61^, ^62^ ^]^ and ensitrelvir^[^ ^63^ ^]^ are being tested. Additionally, existing drugs, such as sodium pyruvate (N115), montelukast (Singulair),^[^ ^64^, ^65^, ^66^, ^67^, ^68^, ^69^, ^70^, ^71^, ^72^, ^73^, ^74^, ^75^, ^76^, ^77^, ^78^, ^79^, ^80^, ^81^, ^82^, ^83^ ^]^ and sirolimus (Rapamune), as well as new drugs, like RSLV‐132 and S‐1226, are undergoing clinical trials to address chronic inflammation associated with long COVID. Furthermore, the metabolic modulator AXA1125 is being tested for its effectiveness in alleviating fatigue, whereas TNX‐102 SL is being tested for its potential to relieve symptoms of long COVID, such as pain. These diverse clinical trials offer hope for effective interventions to alleviate the burden of long COVID.

**Table 3:** Overview of candidate treatments for long COVID in clinical trials.

Drug	Trial	Sponsor	Phase	Enrollment	Primary endpoint	Key eligibility criteria	Pros	Cons	Refs./Trial no:
Nirmatrelvir/Ritonavir (Paxlovid)	STOP‐PASC	Stanford University	2	168	Core Symptoms* Severity Score (Scale 0–3) *fatigue, brain fog, dyspnea, body aches, gastrointestinal and cardiovascular symptoms	At least 2 post‐COVID‐19 symptoms persisted for >3 months (Cases of suspected or confirmed active COVID‐19 infection within 30 days were excluded)	– Multiple trials across institutions enhance data robustness. – Targets major long COVID symptoms like fatigue, brain fog, and dyspnea. – Large sample sizes increase statistical reliability.	Exclusion of active SARS‐CoV‐2 cases limits insights into viral persistence. – Variability in symptom assessment across trials. – Limited long‐term follow‐up.	[91]
	RECOVER‐VITAL	Duke University	2	900	Total number of participants enrolled in each Appendix at day 90 (Symptom Clusters: Exercise intolerance, Cognitive dysfunction, Autonomic dysfunction)	Includes previous suspected, probable, or confirmed COVID‐19(Cases of known active SARS‐CoV‐2 infection within 4 weeks from consent were excluded)	holistic view of long COVID; Large Sample Size, Specific Exclusion Criteria: Excluding those with active SARS‐CoV‐2 infection within 4 weeks ensures the focus is on long‐term recovery, Focus on Real‐World Impact and Potential for Targeted Interventions	Exclusion of Active COVID Cases: Excluding participants with active SARS‐CoV‐2 infection may reduce generalizability. Lack of Control Group: Absence of a control group makes it harder to differentiate long COVID symptoms from other factors, Potential Participant Bias, and Limited Follow‐Up Time.	[92]
	PROLIFIC	Karolinska Institutet	2	400	Change from baseline in quality of life on day 16 (EQ‐5D‐5L VAS scale)	Post‐acute COVID‐19 syndrome (PACS) according to the WHO definition (Type 1, 2 diabetes mellitus, chronic kidney disease, neurodevelopment disorders, active cancer other than localized skin cancer, or immunosuppressive disease were excluded)	Clear Outcome Measure: The study looks at quality of life using a standard test (EQ‐5D‐5L VAS scale) on day 16, Focus on Long‐Term COVID Effects: It focuses on people with Post‐acute COVID‐19 syndrome (PACS), which is important for understanding ongoing COVID effects, Reasonable Sample Size: 400 participants make the results more reliable, **Specific Group of People**: The study picks a clear group of people, making the results easier to interpret, **Important for Public Health**: The study looks at a big health issue, **long‐term COVID**, which is important to understand.	Excludes Certain Health Conditions: People with diabetes, kidney disease, brain disorders, cancer, or weakened immune systems are not included, which may make the results not apply to everyone, **Short Time Frame**: Data is only collected at **day 16**, so it might not show how people feel in the long run, **Might Miss Some People**: By excluding certain groups, the study might not show how PACS affects people with other health issues, **Not Always Representative**: Excluding people with health problems means the study may not apply to everyone who had COVID, **Doesn't Look at All Health Problems**: By excluding people with certain diseases, it might miss how other health conditions affect long COVID.	[93]
	Nirmatrelvir/Ritonavir in Adults	Yale University	2	100	Change in the NIH PROMIS‐29*(a self‐reported 29‐item questionnaire) score on day 28 *depression, physical function, pain interference, fatigue, sleep disturbance etc.	Documented confirmation of previous COVID‐19 infection. Individuals who develop long COVID symptoms 4 weeks after infection, which persist for >12 weeks	Clear Outcome Measure: The study looks at changes in quality of life using a self‐reported questionnaire (NIH PROMIS‐29) at day 28, **Comprehensive Health Areas**: The questionnaire covers **depression, pain, fatigue, sleep, and physical function**, providing a broad picture of health, **Well‐Defined Group**: The study only includes people with a **documented past COVID‐19 infection**, ensuring focused results, **Focus on Long COVID**: It specifically looks at those who develop **long COVID symptoms** 4 weeks after infection, which is an important area of research, **Targeted Treatment**: The study looks at how **Nirmatrelvir/Ritonavir** can affect long COVID symptoms, which could help in developing treatments.	Only Includes Long COVID Patients: The study focuses on people with long COVID (symptoms that last more than 12 weeks), so it may not apply to everyone who had COVID, Short Follow‐Up Period: Data is only collected at day 28, which may not capture long‐term effects of the treatment, **Limited Sample Size**: With only **100 participants**, the study may not capture a diverse range of long COVID experiences, **Self‐Reported Data**: The study relies on self‐reported answers, which can sometimes be biased or inaccurate, **Exclusion of Acute COVID Cases**: Only people with long COVID are included, meaning those still in the acute phase of infection aren't studied.	[94]
	PanoramicNOR	Haukeland University Hospital	3	2000	Presence of the three major long‐COVID symptoms* *fatigue, dyspnea, cognitive symptoms	Individuals who develop COVID‐19 symptoms within 5 days and test positive for COVID‐19 either 2 days before symptom onset or between the onset and randomization	Large Sample Size: The study includes 2000 participants, making the results more reliable and representative, Focus on Key Symptoms: The study looks at the three major long COVID symptoms: fatigue, dyspnea (shortness of breath), and cognitive symptoms, which are very common and important to understand., **Well‐Defined Group**: Participants are clearly defined as those who **test positive for COVID** and show symptoms within a specific time frame, Clear Inclusion Criteria: The study focuses on recently infected individuals, which helps isolate the effects of COVID rather than other factors, Relevant to Ongoing COVID Issues: By focusing on major symptoms, the study directly addresses concerns that impact many COVID survivors.	Only Includes Recent COVID Cases: The study only includes people who test positive within 5 days of symptoms, so it doesn't cover long COVID that develops later, Short Window for Inclusion: The strict time frame (symptoms within 5 days and testing positive just before or after symptoms) may exclude many individuals who have longer or delayed symptom onset, May Miss Other Symptoms: The study focuses on just three symptoms, possibly overlooking other important long COVID symptoms, Limited to Symptomatic COVID Patients: It doesn't include asymptomatic cases or those who tested negative, limiting the study's broader applicability, Excludes Delayed Symptom Development: It may not capture the full picture of long COVID, particularly for those whose symptoms appear later than the study window allows.	[95]
Remdesivir (Veklury)	SOLIDARITY Finland Long‐COVID	Clinical Urology and Epidemiology Working Group	4	202	1) EQ‐VAS (Patient‐reported outcome measure of quality of life on a scale from 0 to 100) [Time Frame: 1 year] 2) EQ‐5D‐5L (Quality of life measure involving five domains, including mobility, self‐care, usual activities, pain/discomfort, and anxiety/depression [Time Frame: 1 year] 3) Recovering from COVID‐19 infection (Five options from “fully recovered” to “not recovered at all”) [Time Frame: 1 year] 4) Fatigue (Scale 0–3) [Time Frame: 1 year] 5) Exertional dyspnea (Scale 0–4) [Time Frame: 1 year] 6) Long COVID symptoms (Infection affected quality of life in the last month as: 0–3) [Time Frame: 1 year]	Patients with confirmed COVID‐19, including those admitted to the ward or intensive care unit (ICU) {Exclusion: patients with severe comorbid conditions with an expected lifespan of less than 3 months as assessed by the investigator, those with ASLT/ALAT levels exceeding 5 times the upper limit of normal, and those with acute comorbid conditions within 7 days prior to participation (excluding troponin elevation due to infection), such as myocardial infarction or unstable angina pectoris}	Phase 4 trial provides a strong evidence base. – Long follow‐up (1 year) for chronic symptom evaluation. – Multi‐domain quality‐of‐life assessments.	– Small sample size for a Phase 4 trial. – Excludes severe comorbidities, limiting generalizability. – Hepatotoxicity concerns due to liver enzyme restrictions.	[96]
Ensitrelvir (Xocova)	PREVAIL‐LC	University of California, San Francisco	2	40	Change in Patient‐Reported Outcomes Measurement Information System (PROMIS)‐29* Score from Baseline physical function, anxiety, depression, fatigue, sleep disturbance, ability to participate in social activities, and pain	At least two moderate symptoms or one severe symptom that are new or worsened since the time of SARS‐CoV‐2 infection	– Covers diverse symptom clusters (fatigue, cognitive dysfunction, sensory issues). – Potential antiviral effect may address viral persistence. – Trials conducted across multiple populations.	– Small sample size in PREVAIL‐LC (40 participants). – Some trials focus only on mild cases, limiting relevance for severe long COVID. – Unclear long‐term mechanism of action.	[64]
	RESILIENCE	Osaka University	–	2000	Proportion of patients with either “any of symptoms of fatigue, shortness of breath or difficulty breathing, smell disturbance, taste disturbance at consecutive 1 and 3 months after the start of treatment” or “any of symptoms of difficulty with concentration and thinking, difficulty reasoning and solving problems, memory loss (short or long term) at 3 months after the start of treatment”	Patients with mild COVID‐19 infection who are expected to start the study drug within 72 h of symptom onset (Exclusion: high risk of developing severe COVID‐19)	Large Sample Size: The study has 2000 participants, which makes the results more reliable, Focus on Important Symptoms: It looks at common long COVID symptoms like fatigue, shortness of breath, smell problems, and memory issues, which are helpful for understanding recovery, Early Treatment: Participants start treatment within 72 h of having symptoms, which is important for early recovery, Clear Symptom Tracking: The study clearly measures specific symptoms at set times, which makes it easier to understand the results, Helps with Long COVID: The study focuses on symptoms that affect people after recovering from COVID, which could lead to better treatments.	Excludes High‐Risk People: People who are at high risk of severe COVID‐19 aren't included, so the study might not apply to them, Short Time for Symptoms: The study only looks at symptoms at 1 and 3 months, which might miss longer‐lasting effects of long COVID, Only Mild Cases: It only includes people with mild COVID‐19, so the results may not apply to people who had more serious illness, Excludes Severe Cases: By excluding high‐risk people, it misses how the treatment might work for more serious cases of COVID‐19, Doesn't Include Complex Cases: It doesn't include people with severe COVID‐19 or other health problems, so the findings may not apply to those groups.	[64]
RSLV‐132	Phase 2 Study of RSLV‐132 in Subjects With Long COVID	Resolve Therapeutics	2	112	PROMIS Fatigue SF 7a T‐score [Time Frame: From Baseline to Day 71] *PROMIS Fatigue SF 7a: Seven questions, measuring symptom severity on a five‐point scale	– Laboratory‐confirmed novel coronavirus (SARS‐CoV‐2) infection as determined by qualitative polymerase chain reaction (PCR) at least 24 weeks prior to baseline – PROMIS Fatigue SF 7a raw score of 21 or greater at screening (confirmed onset of fatigue post‐infection)	– Specifically targets fatigue, a major long COVID issue. – Uses validated PROMIS Fatigue SF 7a assessment. – Potential immune‐modulating effects.	– Small sample size (112 participants). – No combination therapy exploration. – Does not address viral persistence directly.	NCT04944121
N115	Effects of Sodium Pyruvate Nasal Spray in COVID‐19 Long Haulers.	Cellular Sciences, Inc.	2/3	22	1) Change in the symptoms (patient supplied, Scale 0–10) [Time: 14 days] 2) Change in body temperature [Time: 14 days] 3) Change in pulse rate (BPM) [Time: Day 1 (1st day Baseline), Day 8 (8th day Baseline), Day 8 (1st day Post‐treatment), and Day 14 (7th day post‐treatment)] 4)Change in blood oxygenation (%O_2_) [Time Frame: Day 1 (1st day Baseline), Day 8 (8th day Baseline), Day 8 (1st day Post‐treatment), and Day 14 (7th day post‐treatment)]	A prior confirmed positive test for COVID‐19 and lingering symptoms (*CDC website) are required for inclusion *Tiredness/Fatigue, Difficulty thinking/concentrating (Brain fog), Headache, Loss of smell/taste, Dizziness on standing, Fast‐beating/Pounding heart (Heart palpitations), Chest pain, Difficulty breathing/Shortness of breath, Cough, Joint/Muscle pain, Depression/Anxiety, Fever, Symptoms that get worse after physical/mental activities	– Novel nasal delivery may enhance bioavailability. – Targets multiple symptoms, including brain fog and fatigue. – Short trial duration allows rapid results.	– Extremely small sample size (22 participants). – Lacks mechanistic data. – No control group for comparison.	[97]
Montelukast (Singulair)	E‐SPERANZA	Fundacio d'Investigacio en Atencio Primaria Jordi Gol i Gurina	3	284	COP Assessment Test Scale (CAT) (Quality of life based on respiratory symptoms; 8 items, Scale 0–5, Total score 0–40) (Time Frame: 7, 14, 21 and 28 days)	Patients with COVID‐19 with positive CRP within 10 days of symptom onset, including those with persistent respiratory symptoms (lasting more than 1 month but less than 12 months) and mild‐moderate dyspnea (Exclusion: Severe cases)	– Targets persistent respiratory symptoms, a major long COVID concern. – Phase 3 status supports existing evidence. – Safe, widely used asthma medication.	– Excludes severe cases, limiting applicability. – Primarily focuses on respiratory symptoms, not systemic or neurological effects. – Short duration (28 days).	[98]
Deupirfenidone	LYT‐100 in Post‐acute COVID‐19 respiratory disease	PureTech	2	185	Change in distance walked on the 6‐minute walk test (6MWT) [Time Frame: Baseline to Day 91]	Positive molecular RT‐qPCR diagnostic test result or SARS‐CoV‐2 RNA result from a clinical specimen deemed clinically associated with the current episode of illness, warranting hospital admission as per the investigator's judgement, or previously hospitalized (central and/or local laboratory COVID‐19 test results are accepted from any biological material source)	– Addresses post‐acute COVID lung complications. – Uses 6‐minute walk test (6MWT) to assess function. – Targets lung fibrosis, a key issue.	– Small sample size (185 participants). – No assessment of neurological or fatigue‐related symptoms. – Requires prior hospitalization, excluding milder cases.	[99]
Sirolimus (Rapamune)	Assessing the Efficacy of Sirolimus in Patients With COVID‐19 Pneumonia for Prevention of Post‐COVID Fibrosis	University of Chicago	2/3	60	Prevalence of pulmonary fibrosis as evidenced by CT scan (number of patients with >10% pulmonary fibrosis on chest CT) [Time Frame: 12 Weeks]	Hospitalized patients, those diagnosed with COVID‐19 pneumonia, patients with confirmed COVID‐19, and those with less than 10% pulmonary fibrosis (patients previously diagnosed with pulmonary fibrosis or interstitial lung disease were excluded)	– Targets pulmonary fibrosis, a severe complication. – Investigates immunomodulatory potential. – Uses CT imaging for fibrosis assessment.	– Excludes patients with pre‐existing lung disease. – Small sample size (60 participants). – Immunosuppressive effects may pose risks.	NCT04948203
S‐1226	Safety, Tolerability and Efficacy of S‐1226 in post‐COVID‐19 Subjects With Persistent Respiratory Symptoms.	SolAeroMed Inc.	2	48	Treatment‐emergent adverse effects (evaluated as mild, moderate, or severe) [Time Frame: 16 days]	– Prior confirmed COVID‐19 diagnosis by standard RT‐PCR assay or IgM/IgG rapid serological test at least 4 weeks prior to screening visit – Evidence of new and/or persistent respiratory symptoms (cough, wheeze, limitation of activities) at least 4 weeks after the onset of acute COVID‐19 infection	– Focuses on respiratory symptoms, common in long COVID. – Evaluates both safety and efficacy. – Includes real‐world diagnostic methods (PCR, serology).	– Small sample size (48 participants). – Does not address fatigue or neurological dysfunction. – Short duration (16 days).	NCT04949386
Ivabradine (Procoralan)	COVIVA	Uniformed Services University of the Health Sciences	4	250	Change in standing heart rate following 3 months of treatment [Time Frame: 3 months]	Patients with a history of COVID‐19 infection of any severity, those meeting the criteria for long COVID with symptoms persisting beyond 12 weeks after the acute phase, and those not meeting these criteria (cohort separation)	– Targets autonomic dysfunction, a neglected aspect of long COVID. – Phase 4 status ensures prior safety data. – Uses objective standing heart rate changes.	– Limited symptom coverage beyond cardiovascular effects. – Short duration (3 months) may not fully assess long‐term impacts.	[100]
Atorvastatin (Lipitor)	STRONGER	The Geoge Institute	3	400	Neurological Recovery {Processing speed, assessed on the oral Symbol Digit Modalities Test (SDMT)} (Time Frame: 12 months)	History of COVID‐19 confirmed by a positive PCR test, RAT or as per the state guidelines for COVID‐19 diagnosis at the time of screening Any ongoing neurological symptoms as a result of COVID‐19 (e.g., problems with memory, concentration, sleep disturbance and fatigue) that are identified through the administration of the checklist of symptoms on the Somatic and Psychological Health Report (SPHERE) questionnaire, or reported loss of smell (anosmia)	– Investigates neurological recovery, a key issue. – Large sample size (400 participants) for reliability. – Uses SDMT for cognitive function assessment.	– Focuses only on processing speed, missing broader cognitive impacts. – Potential muscle‐related side effects. – Long duration (12 months) delays actionable results.	NCT04904536
TNX‐102 SL	PREVAIL	Tonix Pharmaceuticals, Inc.	2	63	Mean Pain Score (Scale 0–10) [Time Frame: Week 14]	New onset or significant worsening of pain that coincides with a prior COVID‐19 infection and has symptoms present for at least 3 months but no longer than 18 months (Patients with systemic autoimmune diseases are excluded)	– Specifically targets long COVID‐related pain. – Uses validated pain scale (0–10). – Investigates neuroinflammatory mechanisms.	– Small sample size (63 participants). – Excludes patients with autoimmune diseases, limiting relevance. – Moderate trial duration (14 weeks).	NCT05472090
Low dose Naltrexone (Revia)	Low‐dose Naltrexone for Post‐COVID Fatigue Syndrome	Luis Nacul	2	160	Fatigue Intensity (Change in the Fatigue Severity Scale (FSS) total score by 4.7 points or over) [Time Frame: 16 weeks]	(Exclusion: Any use of opioid medications within last 15 days, as reported by the patient)	– Targets post‐COVID fatigue, a major issue. – Uses validated Fatigue Severity Scale (FSS). – Potential neuroimmune effects.	– Excludes opioid users, limiting real‐world applicability. – No secondary endpoints for cognitive or autonomic symptoms. – Small sample size (160 participants).	[101]
AXA1125	Efficacy, Safety, Tolerability of AXA1125 in Fatigue After COVID‐19 Infection	Axcella Health, Inc	2	41	Change from baseline at Week 4 in the phosphocreatine (PCr) recovery rate following moderate exercise, as assessed by 31P‐magnetic resonance spectroscopy (MRS) [Time Frame: Baseline to 28 days]	– Clinically suspected COVID‐19 and a positive antibody test or a documented SARS‐CoV‐2 infection (a positive reverse transcription polymerase chain reaction test) at least 12 weeks prior to screening – Fatigue‐predominant PASC (Exclusion: An explanation for fatigue other than PASC, a medical history that includes non‐invasive or invasive ventilatory support for COVID 19, intensive care unit or other high dependency unit admission for COVID‐19, hospitalization for >1 week for COVID‐19 without intubation)	– Investigates mitochondrial dysfunction in long COVID. – Uses advanced imaging (31P‐MRS) for metabolic analysis. – Focus on fatigue‐predominant PASC.	– Small sample size (41 participants). – Excludes alternative fatigue causes, limiting broad applicability. – Short duration (28 days).	NCT05152849
Temelimab	Temelimab as a Disease Modifying Therapy in Patients With Neuropsychiatric Symptoms in Post‐COVID 19 or PASC Syndrome	GeNeuro SA	2	203	Improvement in fatigue in patients with PASC (defined as a decrease of ≥3 points in the PROMIS Fatigue SF 7a) score, at Week 24 as compared to baseline) [Time Frame: 24 weeks]	– PASC syndrome in accordance with NICE criteria with neuropsychiatric symptoms still occurring >12 weeks after their first appearance – PROMIS Fatigue SF 7a total raw score of ≥21 with onset of fatigue post‐coronavirus disease 2019 (COVID‐19)	– Targets neuropsychiatric symptoms, a major unmet need. – ‐ Uses PROMIS Fatigue SF 7a assessment. – Investigates potential disease‐modifying effects.	– Small sample size (203 participants). – Limited mechanistic data on neuroinflammation modulation. – Short trial duration (24 weeks).	NCT05497089
Vortioxetine	Brain and Cognition Discovery Foundation	Brain and Cognition Discovery Foundation	2	200	Baseline‐to‐endpoint (i.e., Week 8) change in Digit Symbol Substitution Test (DSST) [Time Frame: Weeks 0‐8]	Meets WHO‐defined post‐COVID‐19 condition (Exclusion: Current symptoms are fully explained by major depressive disorder or bipolar disorder)	Clear Outcome Measure: The study uses the Digit Symbol Substitution Test (DSST) to measure changes in cognitive function, which is a well‐established test, Short Time Frame: The study measures changes between Week 0 and Week 8, giving a relatively quick view of how well the treatment works, Focus on Post‐COVID‐19 Symptoms: It specifically focuses on people with post‐COVID‐19 condition, which is highly relevant for current health concerns, Targeted Treatment: The study is testing Vortioxetine, which could help with cognitive symptoms in post‐COVID‐19 patients, Relatively Small Sample Size: With 200 participants, the study can still provide useful insights while being easier to manage and monitor.	Excludes Some Mental Health Conditions: People with current symptoms explained by major depressive disorder or bipolar disorder are not included, which may limit the findings for those with both conditions, Limited Time for Change: The 8‐week period may not be long enough to see long‐term effects or full recovery, Exclusion of Mental Health Causes: Excluding people whose symptoms are explained by mental health conditions might leave out those who have overlapping issues with long COVID, Narrow Inclusion Criteria: By excluding people with specific mental health disorders, the study may miss a wider range of post‐COVID experiences, Potential Bias from Exclusion: Excluding those with depression or bipolar disorder may limit how representative the study sample is of the general post‐COVID population.	[102]

## 5 Niclosamide for Long COVID

As discussed previously, the clinical trials addressing long COVID are predominantly centered on two primary therapeutic approaches: antiviral drugs aimed at targeting the persistent viral presence, and anti‐inflammatory drugs intended to mitigate the inflammation that significantly contributes to long COVID symptoms. This dual focus is driven by the recognition that long COVID, or post‐acute sequelae of SARS‐CoV‐2 infection (PASC), is a multifaceted condition. The persistence of the virus in the body, along with the subsequent inflammatory responses, are key factor underpinning the ongoing symptoms and health issues faced by individuals who have recovered from the initial acute phase of COVID‐19 but continue to experience lingering effects.

Antiviral drugs play a crucial role in addressing the viral aspect of long COVID. The idea is that by targeting and eliminating residual viral particles or preventing their replication, these drugs could potentially alleviate symptoms and contribute to recovery. Various antiviral agents have been tested in clinical settings, focusing on reducing viral load and preventing viral persistence, which is believed to play a significant role in the development and continuation of long COVID symptoms. The effectiveness of these antiviral drugs is measured by their ability to reduce the viral reservoir in the body, thus potentially decreasing the duration and severity of long COVID manifestations. On the other hand, the anti‐inflammatory approach targets the inflammation that persists even after the acute phase of the infection has resolved. Chronic inflammation is thought to be a significant contributor to the ongoing symptoms of long COVID. Anti‐inflammatory drugs aim to modulate the immune response and reduce the inflammatory markers and cytokines that are involved in sustaining these symptoms. By addressing the inflammatory component, these drugs seek to improve the quality of life for long COVID patients by reducing the inflammatory burden and alleviating associated symptoms.^[^ ^84^ ^]^

In this context, niclosamide has emerged as a promising candidate for long COVID treatment. Although niclosamide was clinically approved for treating parasitic infections in humans, its poor aqueous solubility has presented significant challenges, as previously mentioned. This limitation has restricted its broader therapeutic use, despite its potential in other areas, including antiviral and anticancer therapies. However, research has uncovered its potential beyond its initial application. Niclosamide has demonstrated both antiviral and anti‐inflammatory properties (Figure 1), making it an intriguing option for long COVID therapy. The antiviral activity of niclosamide is notable in its ability to interfere with the replication of viruses. This mechanism is particularly relevant in the context of long COVID, where residual viral particles may contribute to the persistence of symptoms.^[^ ^85^ ^]^ Niclosamide's action on viral replication could help in reducing the viral load and potentially addressing the underlying cause of ongoing symptoms. Moreover, niclosamide's anti‐inflammatory effects are equally significant. The drug has been shown to influence various inflammatory pathways and cytokines, potentially reducing the chronic inflammation associated with long COVID. By targeting inflammation, niclosamide could help in alleviating symptoms such as fatigue, pain, and cognitive dysfunction, which are common among long COVID patients and are thought to arise from underlying mechanisms—such as chronic inflammation, immune dysregulation, and neuroglial activation—that are also implicated in various other neurological and neuroinflammatory disorders. Thus, clinical trials for long COVID are exploring both antiviral and anti‐inflammatory strategies to address the complex nature of this condition. Niclosamide stands out due to its dual‐action capabilities, offering a potential treatment that could target both the persistent viral elements and the chronic inflammation associated with long COVID. As research progresses, niclosamide's efficacy and safety as a treatment option will become clearer, potentially providing a valuable therapeutic avenue for managing and mitigating the impacts of long COVID.

## 6 Antiviral Activity of Niclosamide

The antiviral effects of niclosamide against SARS‐CoV‐2 have been demonstrated through screening studies and other approaches. Jeon et al. screened 48 FDA‐approved drugs for antiviral efficacy against SARS‐CoV‐2 and identified 24 potential antiviral drugs. Among these, niclosamide and ciclesonide demonstrated notable antiviral effects. Niclosamide, a drug long used as an anthelmintic, exhibited potent antiviral activity against SARS‐CoV‐2 (IC50 = 0.28 µM).^[^ ^6^ ^]^ The mechanism by which niclosamide exerts its antiviral effect against SARS‐CoV‐2 was elucidated by Gassen et al. Following their studies on the antiviral mechanism against MERS‐CoV, it is found that niclosamide inhibits SKP2 and stabilizes BECN1, inducing autophagy, which in turn suppresses SARS‐CoV‐2.^[^ ^86^ ^]^ Furthermore, this antiviral efficacy of niclosamide is maintained regardless of the SARS‐CoV‐2 variants, with no significant differences among IC50 values against alpha (B.1.1.7), beta (B.1.351), and delta (B.1.617.2) variants.^[^ ^87^ ^]^

Another plausible mechanism underlying the antiviral activity of niclosamide is its endosomal pH‐neutralizing effect. The replication processes of the influenza virus and rhinovirus depend on endo‐lysosomal pH, and niclosamide inhibits viral replication by neutralizing the endo‐lysosomal pH.^[^ ^8^ ^]^ Additionally, niclosamide inhibits the replication of the chikungunya virus, which enters cells via receptor‐mediated endocytosis.^[^ ^88^ ^]^ It also inhibits Zika virus replication and the resulting cell death by attenuating pH‐dependent membrane fusion.^[^ ^89^ ^]^

## 7 Anti‐Inflammatory Activity of Niclosamide

Niclosamide has anti‐inflammatory and immune‐regulatory activities, making it promising for treating long COVID by suppressing excessive inflammation. It was identified as the best hit agent for inhibiting inflammation through inflammasome suppression in a study that screened 2560 drugs.^[^ ^102^ ^]^ Additionally, inflammation caused by SARS‐CoV‐2 is related to the activation of TMEM16 family members by the spike protein. For example, TMEM16A promotes NF‐κB activation and IL‐6 secretion, while TMEM16F is involved in blood coagulation and thrombosis.^[^ ^103^, ^104^, ^105^ ^]^ Previous studies have found that niclosamide is a potent inhibitor of the Ca^2+^‐activated Cl‐ channel TMEM16A, which is found in airway smooth muscles and plays a role in bronchoconstriction and thus increases mucus production in chronic inflammatory airway diseases.^[^ ^106^, ^107^ ^]^ Miner et al. demonstrated that niclosamide has a beneficial effect on constricted airways and mucus hypersecretion during airway inflammation by inhibiting airway TMEM16A.^[^ ^108^ ^]^ Furthermore, niclosamide inhibits the progression of asthma, chronic obstructive pulmonary disease (COPD), and cystic fibrosis by suppressing the release of IL‐8.^[^ ^106^ ^]^ Braga et al. discovered that niclosamide markedly suppresses the activity of TMEM16F, thereby reducing calcium fluctuations and membrane conductivity in cells expressing spike proteins.^[^ ^109^ ^]^

## 8 Hurdles in Repurposing Niclosamide as a Treatment for Long COVID

Long COVID, characterized by persistent fatigue, respiratory issues, cognitive dysfunction, and other symptoms, currently lacks definitive treatment. Niclosamide, an established anthelmintic drug, has emerged as a potential candidate for the treatment of long COVID, given its antiviral and anti‐inflammatory properties. However, several hurdles must be addressed to successfully repurpose niclosamide.

### 8.1 Pharmacokinetic Challenges

**Figure 2:**
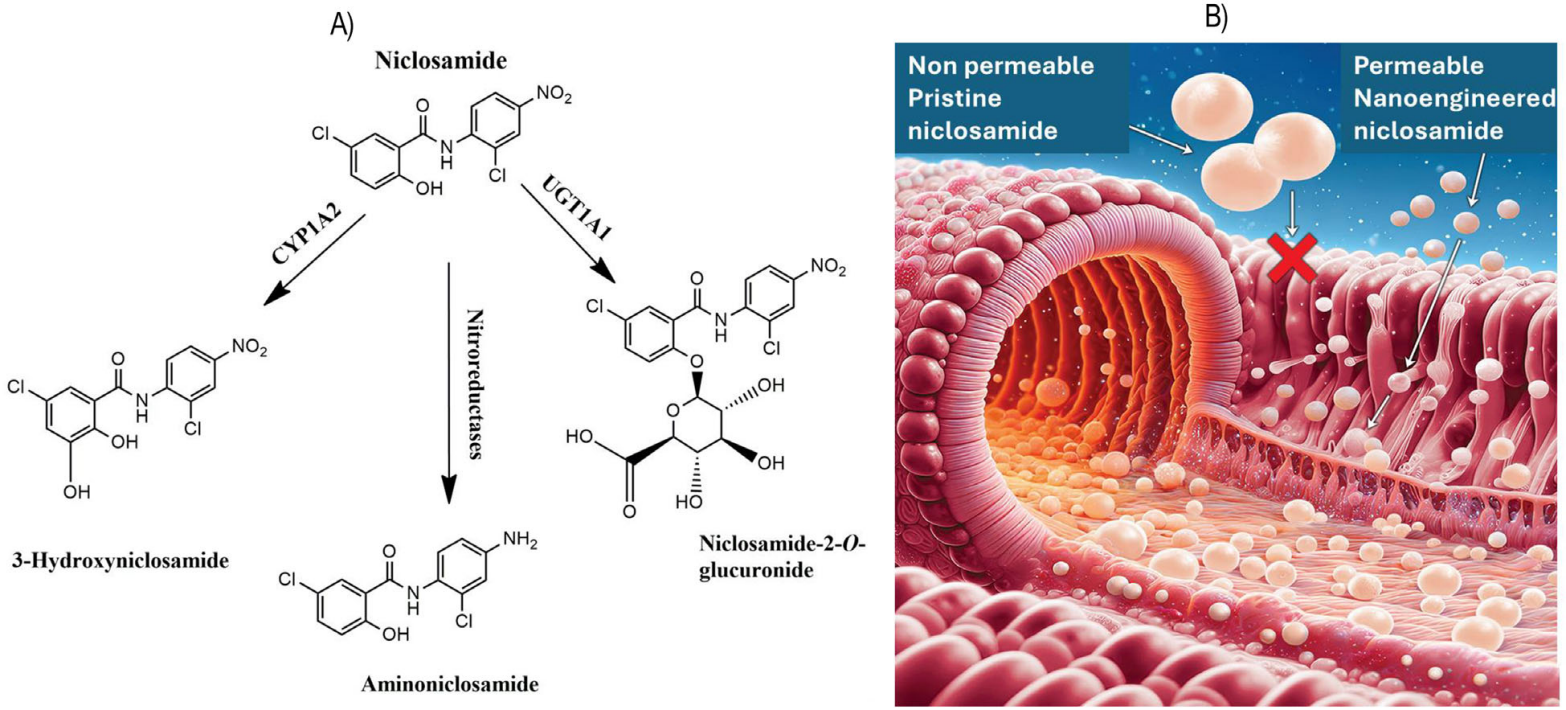
A) Metabolism and B) membrane permeation of niclosamide through nanoengineered niclosamide (CYP1A2‐Cytochrome P450 1A2, a member of the cytochrome P450; UGT1A1‐ bilirubin uridine diphosphate glucuronosyl transferase (bilirubin‐UGT) enzyme); (Image was created using Microsoft Image creator Bing/adobe photoshop).

One of the primary hurdles in repurposing niclosamide for long COVID is its pharmacokinetic profile.^[^ ^110^ ^]^ Niclosamide has a very low aqueous solubility of 0.23 µg mL^−1^, which results in poor bioavailability (≈10%) when administered orally, meaning that only a small amount of the drug reaches systemic circulation.^[^ ^111^ ^]^ This limits its effectiveness in treating conditions that require systemic action rather than localized action in the gut (e.g., against parasitic worms). Additionally, the fact that niclosamide is rapidly metabolized (mainly into three metabolites, such as 3‐hydrixy niclosamide, amino niclosamide, and niclosamide‐2‐*O*‐glucuronide) in the intestines and liver is another factor hindering its repurposing (**Figure** **2**). Fan et al. discovered that both hepatic glucuronidation and intestinal ones have a more significant influence on the metabolic pathway of orally administered niclosamide than the P450‐mediated metabolic pathway.

They found that niclosamide is quickly metabolized through glucuronidation in the liver and intestines. Therefore, they proposed a strategy of inhibiting niclosamide glucuronidation in both the liver and intestines to improve its bioavailability.^[^ ^112^ ^]^ However, enhancing drug bioavailability by inhibiting metabolic enzymes can lead to drug‐drug interactions (DDIs), requiring a cautious approach. Such a careful strategy can be achieved by preventing the breakdown of a drug into inactive or less active metabolites, allowing the drug to remain in its active form for longer periods, increasing its therapeutic effect, and reducing the need for higher doses. By limiting metabolism, the risk of DDIs‐where one drug accelerates or diminishes the metabolism of another drug, leading to reduced efficacy or increased toxicity, is minimized. However, from a safety perspective, it is recommended to enhance niclosamide bioavailability through methods other than enzyme inhibition.

### 8.2 Dosage and Administration

Determining the appropriate dosage and administration regimen for niclosamide is a crucial factor when conducting clinical trials for long COVID. The development of formulations that enhance niclosamide's bioavailability will influence the amount and dosage required for treatment. Additionally, the treatment duration for long COVID will likely need to differ from that for acute SARS‐CoV‐2 infection. For example, while Paxlovid is administered for 5 days to treat acute SARS‐CoV‐2 infection, in clinical trials for long COVID (NCT05823896),^[^ ^113^ ^]^ it is administered for 15 days. Similarly, treating long COVID may require a longer administration period compared to acute viral infections. Therefore, it will be necessary to demonstrate that the drug can be administered safely over a prolonged period, supported by GLP (Good Laboratory Practice) toxicity studies.

## 9 Challenges and Opportunities in Repurposing Niclosamide for Long Covid: A Comprehensive Perspective

Repurposing niclosamide for long COVID presents a range of regulatory, clinical, manufacturing, and economic considerations that must be addressed to facilitate its adoption in real‐world healthcare settings. While niclosamide holds promise due to its broad‐spectrum antiviral properties and known safety profile, significant challenges remain in securing regulatory approval, designing effective clinical trials, ensuring large‐scale production, and overcoming economic barriers.

### 9.1 Regulatory Approvals and Policy Considerations

Unlike newly developed drugs, repurposing an existing medication involves navigating a unique regulatory pathway. Although niclosamide is already approved as an anthelmintic, securing approval for long COVID treatment requires robust clinical trial data to demonstrate both efficacy and safety for this novel indication. Regulatory agencies, such as the U.S. FDA and the European Medicines Agency (EMA), require evidence from well‐designed trials before granting authorization.^[^ ^114^ ^]^ However, given its long history of human use and established safety profile, regulatory frameworks that expedite approvals for repurposed drugs could be leveraged to streamline the process. Adaptive regulatory strategies, such as conditional approvals or emergency use authorizations, could also accelerate access to niclosamide while further data is gathered.

### 9.2 Optimizing Clinical Trial Designs for Niclosamide in Long COVID

Long COVID is a heterogeneous condition with a wide spectrum of symptoms, ranging from persistent fatigue and respiratory issues to neuropsychiatric manifestations like brain fog and cognitive dysfunction. Designing an effective clinical trial for niclosamide must take this variability into account by selecting appropriate endpoints that reflect meaningful improvements in patient health. Symptom alleviation and quality‐of‐life enhancement could serve as primary clinical endpoints, alongside biomarkers that indicate reduced inflammation and viral persistence.

A key factor in trial design is ensuring adequate statistical power, which requires careful determination of the sample size. Investigator‐led clinical trials, pragmatic trials, or adaptive trial designs could be employed to refine treatment strategies dynamically. Additionally, the ability of niclosamide to cross the blood‐brain barrier makes it particularly relevant for addressing neuropsychiatric symptoms linked to vascular inflammation and neuronal damage caused by SARS‐CoV‐2.^[^ ^56^ ^]^ Trials could therefore focus on its potential neuroprotective effects, providing a distinct therapeutic advantage over other treatments that do not effectively reach the central nervous system.

### 9.3 Manufacturing Scalability and Supply Chain Challenges

Scaling up niclosamide production to meet increased demand poses several logistical hurdles. Traditional formulations of niclosamide suffer from poor bioavailability, necessitating the development of optimized nanoengineered formulations to enhance absorption and therapeutic efficacy. However, manufacturing advanced formulations at a commercial scale requires significant infrastructure investment and compliance with stringent quality control measures.^[^ ^115^ ^]^

Furthermore, the global pharmaceutical supply chain has faced considerable disruptions due to the COVID‐19 pandemic, which could impact the consistent availability of niclosamide. Establishing a stable supply chain, diversifying production sites, and securing raw materials are critical steps to ensure uninterrupted access. Collaborations between government agencies, pharmaceutical companies, and contract manufacturing organizations (CMOs) could help address these challenges and facilitate large‐scale production.

### 9.4 Economic and Funding Barriers in Drug Repurposing

One of the primary economic challenges in repurposing niclosamide is the lack of financial incentives for pharmaceutical companies. Since niclosamide is off‐patent, there is little commercial motivation for private sector investment in clinical trials and large‐scale production. Without strong patent protection, companies may struggle to recoup research and development costs, leading to reluctance in funding further investigations.

To overcome this barrier, public funding, non‐profit initiatives, and government‐backed research programs play a crucial role. International collaborations between academic institutions, health agencies, and philanthropic organizations could support the necessary clinical trials and facilitate equitable access to niclosamide if proven effective. Governments may also consider market incentives, such as extended exclusivity for new formulations or reimbursement policies that encourage adoption.

While niclosamide presents a compelling case for repurposing as a treatment for long COVID, multiple real‐world challenges must be addressed to bring it into widespread clinical use. Regulatory hurdles, trial design complexities, production scalability, and economic disincentives require coordinated efforts among policymakers, researchers, and industry stakeholders. By strategically addressing these challenges, niclosamide could emerge as an accessible, cost‐effective, and potentially transformative option for patients suffering from long COVID, bridging the gap between scientific innovation and practical healthcare implementation.

## 10 Overcoming Niclosamide Pharmacokinetic Challenges

To overcome these pharmacokinetic hurdles, several strategies can be employed, as described in **Table** **4**.

**Table 4:** Various approaches for addressing the pharmacokinetic challenges of niclosamide.

Formulation development			
Formulation	Components	Key points	Refs.
Solid Lipid Nanoparticles (SLNs)	Egg phosphatidylcholine (Egg PC), cholesterol, distearoyl phosphatidylethanolamine (DSPE)‐PEG1000 and DSPE‐PEG750	Encapsulating niclosamide in SLNs can improve its stability and absorption in the gastrointestinal tract, thereby increasing its bioavailability.	[117]
Liposomal Formulations	Liposome	Liposomes can encapsulate niclosamide, protecting it from degradation in the stomach and enhancing its absorption through the intestinal wall.	[118]
Cyclodextrin Complexes:	4‐Sulphonato‐calix[n]arenes and cyclodextrins	Forming inclusion complexes with cyclodextrins can enhance niclosamide solubility and bioavailability.	[119]
Inorganic Nanoparticles such as MgO	Magnesium oxide, Hydroxy propyl methyl cellulose	Improved PK and anti‐COVID effects	
Polymeric Micelles		These can improve the solubility and stability of niclosamide, facilitating better absorption and distribution in the body.	[120]
Prodrug Approaches			
Converting niclosamide into a prodrug	Niclosamide analogues	Enhanced solubility and permeability can be obtained. Prodrugs are inactive derivatives that can be metabolized into the active drug within the body, potentially improving bioavailability and pharmacokinetics.	[121]
Nanotechnology‐Based Delivery Systems			
Nanoparticles: Utilizing nanoparticles for drug delivery	Organic, inorganic and hybrid nanoparticles can be used	Nanoparticles can enhance the solubility, stability, and bioavailability of niclosamide and provide targeted delivery, reducing systemic side effects	[122]
Nanocrystals	Spray‐dried microparticles	Reducing niclosamide to nanocrystal form can increase its surface area and dissolution rate, leading to better absorption and bioavailability.	[123]
Co‐administration with Absorption Enhancers			
Bioenhancers	Piperine	Co‐administering niclosamide with substances that enhance absorption, such as piperine or certain fatty acids, can improve its bioavailability by increasing permeability across the intestinal lining.	[124]
Permeation Enhancers	Hydroxyethyl cellulose	Substances can temporarily disrupt the tight junctions between epithelial cells in the gastrointestinal tract, facilitating better absorption of niclosamide.	[125]
Pharmacokinetic Modeling and Optimization			
Computational Models	Using pharmacokinetic and pharmacodynamic modeling can help predict the behavior of niclosamide in the body and guide the design of optimized formulations and dosing regimens.		[126]
Optimized Dosing Strategies	Adjusting the dosing frequency and amount based on pharmacokinetic data can help maintain therapeutic levels of niclosamide in the bloodstream, improving its efficacy against long COVID.		[127]

## 11 Nanoengineered Niclosamide as a Solution for Long COVID

Niclosamide has faced a 60‐year conundrum in its application due to pharmacological challenges. Originally developed as an antiparasitic drug, its poor solubility and limited bioavailability restricted its effectiveness to intestinal parasitic infections. Despite demonstrating promising antiviral and anticancer potential in laboratory settings, its rapid metabolism and poor systemic absorption have hindered broader applications.

To overcome the limitation of niclosamide's low bioavailability, researchers have applied nano‐engineering technologies. Lin et al. developed nanosized niclosamide using electrospray technology. The nanosized niclosamide they developed (nano‐NI colloidal dispersion) had an average particle diameter and length of 105−21 and 493−151 nm, respectively.^[^ ^111^ ^]^ Upon oral administration in rats, enhanced bioavailability was confirmed. Jara et al. enhanced the bioavailability of niclosamide through amorphous niclosamide. The niclosamide formulation they developed, which mixed PVP‐VA and TPGS, increased oral bioavailability of niclosamide by 2.6‐fold, as confirmed in experiments using Sprague‐Dawley rats. Gan et al. prepared niclosamide nanoparticles using the solvent evaporation method with PCEC (poly(ɛ‐caprolactone, ɛ‐CL)‐poly(ethylene glycol)‐poly(ɛ‐CL)) and SDS (Sodium dodecyl sulfate).

These particles had an average size of ≈172 ± 2 nm and exhibited improved water solubility.

However, these achievements have only reached the preclinical study stage and have not progressed to clinical trials. To enter clinical trials, CMC (Chemistry, Manufacturing, and Controls) studies to support large‐scale production, and GLP (Good Laboratory Practice) toxicity studies to ensure human safety, are required.^[^ ^127^ ^]^

By meeting these requirements and capitalizing on the surged interest in drug repositioning during the recent COVID‐19 pandemic, a formulation that overcame niclosamide's low bioavailability was extended to clinical trials for COVID‐19 treatment.

**Figure 3:**
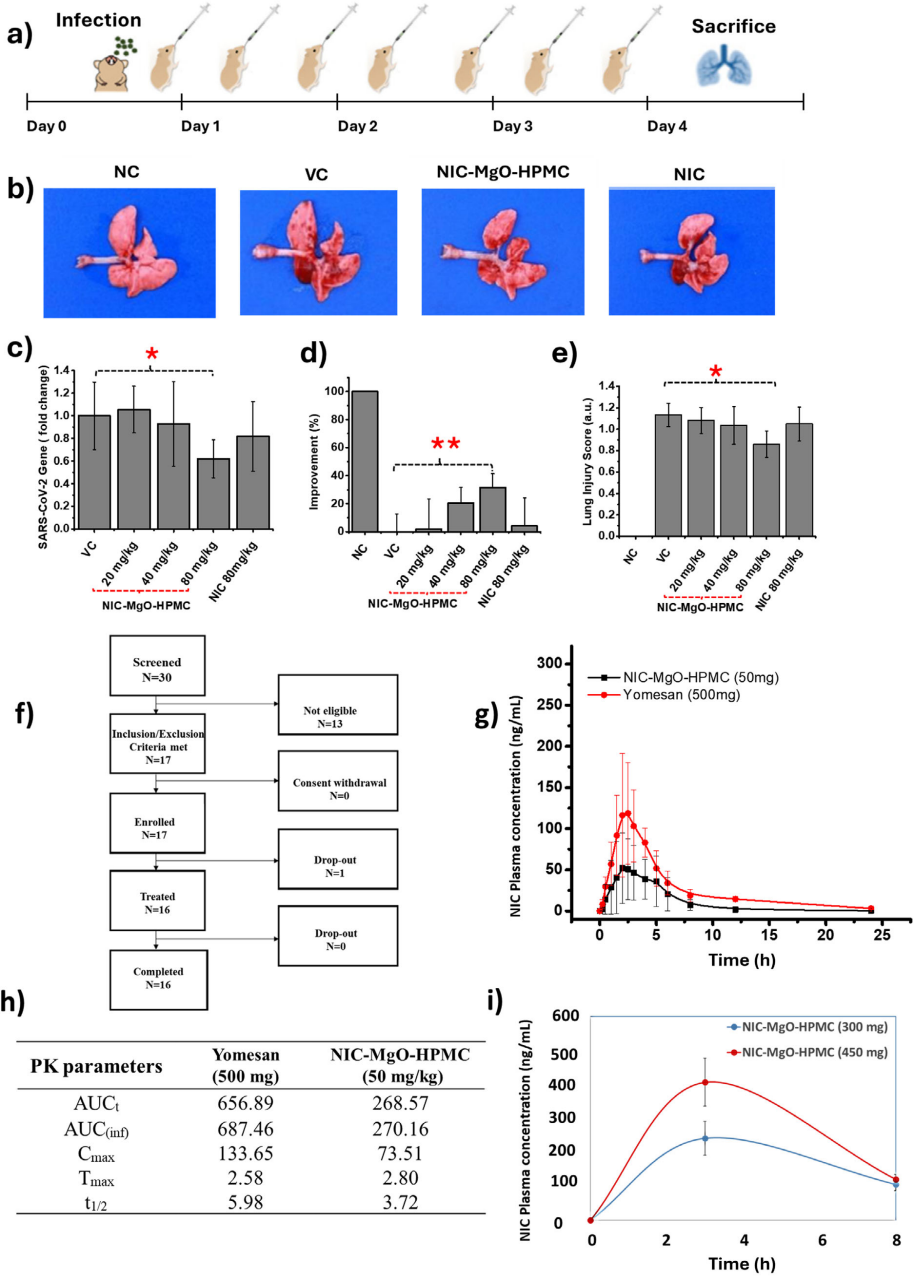
Therapeutic activity of NIC–MgO–HPMC observed in the golden Syrian hamster model. a) Hamsters were intranasally infected with SARS‐CoV‐2. NIC–MgO–HPMC was administered postinfection starting 6 h postinfection. The samples were administered every 12 h for 4 days. Hamsters were euthanized on 4 dpi, and their lungs were harvested. b) One representative image of the lung was chosen for each group. c) Lung viral loads were determined by using quantitative reverse transcription polymerase chain reaction (RT‐qPCR). d) Gross pathological lung lesion. At necropsy, the percentage of each lung lobe affected by gross lesions was estimated. e) Histopathological examination of the lungs of a hamster model. Lung samples were stained with H&E and analyzed for the presence of a lesion. f) Phase 1 subject dispositions g), PK profiles, and h) PK parameters of Phase 1 clinical trial result of NIC‐MgO‐HPMC and Yomesan (six participants received Yomesan and 10 participants received NIC‐MgO‐HPMC capsule, data are presented as means± S.E); i) PK profiles from randomized, double‐blind, placebo‐controlled phase 2 clinical trial for NIC‐MgO‐HPMC with low dose (300 mg, *n* = 20) and high dose (450 mg, *n* = 18) (data are presented as means± S.E). Among the participants in the Phase 2 clinical trial (*n* = 300), only a few of them consented to and participated in blood sampling for the PK study. (Reused with permission from Small^[^ ^128^ ^]^ Copyright 2024, Wiley).

In Choy et al.’s study using an inorganic carrier, nanoengineered NIC‐MgO‐HPMC was shown to significantly reduce viral load in the lungs, the target organ of SARS‐CoV‐2 infection, and suppress inflammation by increasing bioavailability in an animal model (**Figure** **3**). It also demonstrated its improved bioavailability in humans compared to traditional niclosamide during clinical trials.^[^ ^128^ ^]^

Preclinical and clinical studies on this nanoengineered niclosamide highlighted significant advancements in overcoming niclosamide's solubility challenges. Nanosized MgO, a basic solid, creates an alkaline environment on the external surface, where adsorbed hydrophobic niclosamide could be deprotonated and eventually increase its solubility, while HPMC (hydroxypropyl methylcellulose), a hydrophilic polymer, also enhances its dispersion in aqueous environments, promoting sustained release. Together, these components increase solubility and bioavailability, making niclosamide more effective for therapeutic use, especially against viral infections. The study revealed that both traditional niclosamide and its nanoengineered NIC‐MgO‐HPMC share the same metabolic pathways (Figure S1, Supporting Information).^[^ ^128^ ^]^ However, the enhanced intestinal uptake of NIC‐MgO‐HPMC contributes to the improved bioavailability of niclosamide, leading to more effective therapeutic action.

## 12 Prospects for Global Impact and Accessibility

The strategic application of nanoengineered niclosamide offers immense global potential, particularly due to its pre‐existing FDA approval, which could expedite its application as a therapeutic for long COVID. Unlike developing new drugs from scratch, repurposing this known drug provides a unique advantage by bypassing extensive safety profiling, allowing for faster regulatory approvals. Niclosamide's history as a safe antiparasitic agent positions it as a potentially invaluable option for pandemic‐related ailments, both in high‐ and low‐resource settings. The ability to tap into existing manufacturing capabilities reduces the time to market, making it a feasible and scalable solution for mass production, especially with the backing of international health organizations. Further, advancements in nanotechnology‐based formulations can make niclosamide more affordable by optimizing its PK properties, thereby enhancing its bioavailability and therapeutic efficacy while minimizing the required dosage. In many parts of the world where healthcare resources are limited, access to high‐cost medications remains a significant barrier. By employing nanohybridized forms of niclosamide that lower production costs and improve efficiency, pharmaceutical companies can help bridge the gap, ensuring this medication reaches individuals in resource‐constrained regions. With its broad‐spectrum antiviral properties, niclosamide holds potential beyond COVID‐19, providing a therapeutic that could be deployed against multiple viral threats, a benefit in pandemic preparedness. Furthermore, its immunomodulatory and anti‐inflammatory properties suggest broad applications beyond viral infections. Niclosamide's ability to interact with multiple signaling pathways could play a role in managing other chronic inflammatory and immune‐related conditions. For global health, this implies a dual‐purpose drug, one that addresses both infectious disease and chronic inflammatory states that can arise in post‐viral conditions. Global health strategies can thus adopt niclosamide as a multipurpose, adaptable drug, which could significantly alleviate healthcare burdens worldwide, especially during future viral outbreaks.

## 13 Future Directions and Collaborative Research

The successful repositioning of niclosamide and nanoengineered one for long COVID hinges on interdisciplinary collaboration among scientists across pharmacology, nanotechnology, molecular biology, and clinical medicine. While its efficacy in parasitic infections has been well‐documented, maximizing its potential for viral infections, especially long COVID, demands optimized dosing strategies and formulations tailored to this new application. Such efforts will necessitate researchers across disciplines to refine nanohybridization methods that maximize bioavailability and enhance targeted delivery. By engineering nanoparticle‐based delivery vehicles that encapsulate niclosamide, researchers can control the drug's release and target specific tissues, reducing systemic exposure and minimizing various side effects.

In addition to enhancing formulation and delivery techniques, researchers must explore the potential of combination therapies that use nanoengineered niclosamide in tandem with other therapeutic agents. Given the complex pathology of long COVID, which affects multiple organ systems, a multi‐targeted therapeutic approach may be necessary. Collaborative research could focus on pairing nanoengineered niclosamide with other anti‐inflammatory or immune‐modulating agents, potentially achieving synergistic effects that enhance patient outcomes. Preclinical studies testing these combination strategies could offer valuable insights into therapeutic efficacy, providing a foundation for subsequent clinical trials.

An essential area of exploration will be niclosamide's potential to inhibit pathways central to viral replication and inflammation. Investigating its effects on viral entry points, replication, and immune response can yield data necessary to develop tailored treatment plans that specifically target long COVID at a molecular level. By conducting in vitro and in vivo studies on its effects in animal models, researchers can gain insights into how nanoengineered niclosamide interacts with human cellular pathways, setting the groundwork for well‐designed human trials. Such studies should also explore optimal dosing, investigating whether nanoengineered niclosamide's effects can be enhanced with lower, safer doses when delivered in a nanohybridized form.

To further validate its use, longitudinal studies will be critical to understanding niclosamide's efficacy over time in long COVID patients. Monitoring patient outcomes over extended periods can offer data on symptom relief, inflammatory markers, and quality of life, allowing researchers to fine‐tune therapeutic approaches. These studies could also include health economics analyses to understand the broader impacts of niclosamide on healthcare systems, particularly in terms of reducing long‐term medical costs and improving workforce productivity by alleviating chronic post‐COVID symptoms.

## 14 Perspective: Integrating Nanoengineered Niclosamide within the Evolving Landscape of Long Covid Management

Long COVID presents a persistent global health challenge, with symptoms affecting multiple organ systems and significantly impairing quality of life. Despite extensive research efforts, no definitive treatment has been established, highlighting the need for a multidimensional therapeutic approach. A comprehensive understanding of long COVID pathophysiology, the current treatment landscape, and ongoing clinical trials forms the foundation for identifying promising therapeutic strategies. By contextualizing emerging candidates within this broader framework, a balanced discussion can be established that evaluates their potential alongside existing and investigational treatment approaches.

Among the various therapeutic candidates, nanoengineered niclosamide has gained attention due to its antiviral, anti‐inflammatory, and immunomodulatory properties. However, rather than being positioned as a standalone treatment, it is best considered as an emerging option within the broader scope of long COVID management. Given the multifaceted nature of long COVID pathogenesis—encompassing viral persistence, immune dysregulation, endothelial dysfunction, and metabolic disturbances—a combination of targeted interventions may be necessary. This perspective allows for an assessment of nanoengineered niclosamide as a complementary therapeutic strategy, either alone or in synergy with other treatments, rather than as the central focus of long COVID therapy.

Despite its promising pharmacological profile, niclosamide faces several translational challenges. Clinical evidence supporting its efficacy in long COVID remains limited, as most trials have focused on acute SARS‐CoV‐2 infection rather than post‐viral syndromes. Additionally, its poor systemic bioavailability, rapid metabolism, and solubility constraints hinder its direct clinical application. Nanoengineering strategies offer potential solutions by improving pharmacokinetics, targeted delivery, and controlled release, yet further validation through rigorous preclinical and clinical studies is essential. Regulatory complexities associated with repurposing niclosamide in a nanoengineered formulation also require careful consideration to ensure clinical feasibility.

Future research should prioritize well‐controlled clinical trials that explore the impact of nanoengineered niclosamide beyond its antiviral potential, assessing its effects on inflammation and immune dysregulation — key pathological drivers of long COVID. Investigating potential synergies with other therapeutic agents may further enhance treatment efficacy, particularly in addressing the multifaceted nature of the disease. Additionally, longitudinal studies will be critical for evaluating long‐term safety, patient outcomes, and real‐world effectiveness.

By integrating nanoengineered niclosamide within the broader investigative landscape of long COVID, a scientifically grounded and clinically relevant evaluation can be achieved. This approach ensures that emerging therapeutics are critically assessed within the evolving paradigm of long COVID management, fostering a more strategic and evidence‐based path toward innovative treatment solutions.

## 15 Conclusion

The journey to repurpose niclosamide as nanoengineered niclosamide for long COVID treatment reflects an innovative path in antiviral therapy, bridging decades of pharmaceutical experience with modern advances in drug delivery. Nanotechnology, specifically, plays a pivotal role in enhancing niclosamide's pharmacokinetics, potentially transforming it from an insoluble and hydrophobic antiparasitic agent to a versatile antiviral therapeutic with broad applications. With techniques such as nanoencapsulation, inorganic nanohybridization, the creation of solid lipid nanoparticles, and the use of biocompatible polymer coatings, the limitations associated with niclosamide's bioavailability and systemic absorption could be overcome. These advances pave the way for clinical applications that are both practical and impactful, particularly in addressing complex diseases like long COVID that lack standardized treatments.

Moving forward, comprehensive clinical trials are necessary to evaluate niclosamide's safety and efficacy specifically for long COVID. By assessing its effects on inflammation, viral load, and multi‐system symptoms, the medical community can determine the viability of this repurposed drug as a long‐term solution for post‐viral syndromes. If successful, nanohybridized niclosamide could serve as an accessible and effective treatment option, contributing to a global strategy aimed at mitigating the ongoing impacts of the COVID‐19 pandemic.

The scalability and affordability of niclosamide could also enable its inclusion in pandemic preparedness plans, offering a versatile treatment option that could be rapidly deployed in case of future viral outbreaks. As the research community continues to unlock the full therapeutic potential of niclosamide, the development of targeted, nanotechnology‐based formulations will be essential to ensuring it can meet the demands of both current and future global health challenges.

## Conflict of Interest

The authors declare no conflict of interest.

## Author Contributions

Each author played a pivotal role in shaping the final version of this review, ensuring a comprehensive and balanced overview of the subject matter.

## Supporting information



Supporting Information
