# Supplementary material for: Transforming Niclosamide through Nanotechnology: A Promising Approach for Long COVID Management
Source: Small. 2025 May 19;21(27):2410345. doi: 10.1002/smll.202410345 (PMC12243710; doi:10.1002/smll.202410345)
Supplement: Supplementary file 1 — Supporting Information [file SMLL-21-2410345-s001.docx]

**Transforming Niclosamide through Nanotechnology: A Promising Approach for Long COVID Management**

*N. Sanoj Rejinold, Goeun Choi, Geun-woo Jin, Jin-Ho Choy^,^*[***](https://pubs.acs.org/action/doSearch?field1=Contrib&text1=%22Andrzej+M.+Z%CC%87ak%22&field2=AllField&text2=&publication=&accessType=allContent&Earliest=&ref=pdf)

| **N. Sanoj Rejinold, Goeun Choi**  Intelligent Nanohybrid Materials Laboratory (INML), Department of Chemistry, School of Science and Technology, Dankook University, Cheonan 31116, Republic of Korea  E-mail: sanojrejinold@dankook.ac.kr orcid.org/0000-0002-7045-0563; E-mail: goeun.choi@dankook.ac.kr  **Geun-woo Jin**  R&D Center, Hyundai Bioscience Co. LTD., Seoul, 07990 Republic of Korea; E-mail: geunwoo.jin@hyundaibio.com  **Jin-Ho Choy**  Intelligent Nanohybrid Materials Laboratory (INML), Department of Chemistry, School of Science and Technology, Dankook University, Cheonan 31116, Republic of Korea  Division of Natural Sciences, The National Academy of Sciences, Seoul 06579, Republic of Korea  orcid.org/0000-0002-4149-7100  *Corresponding author: **jhchoy@dankook.ac.kr** |
| --- |

**
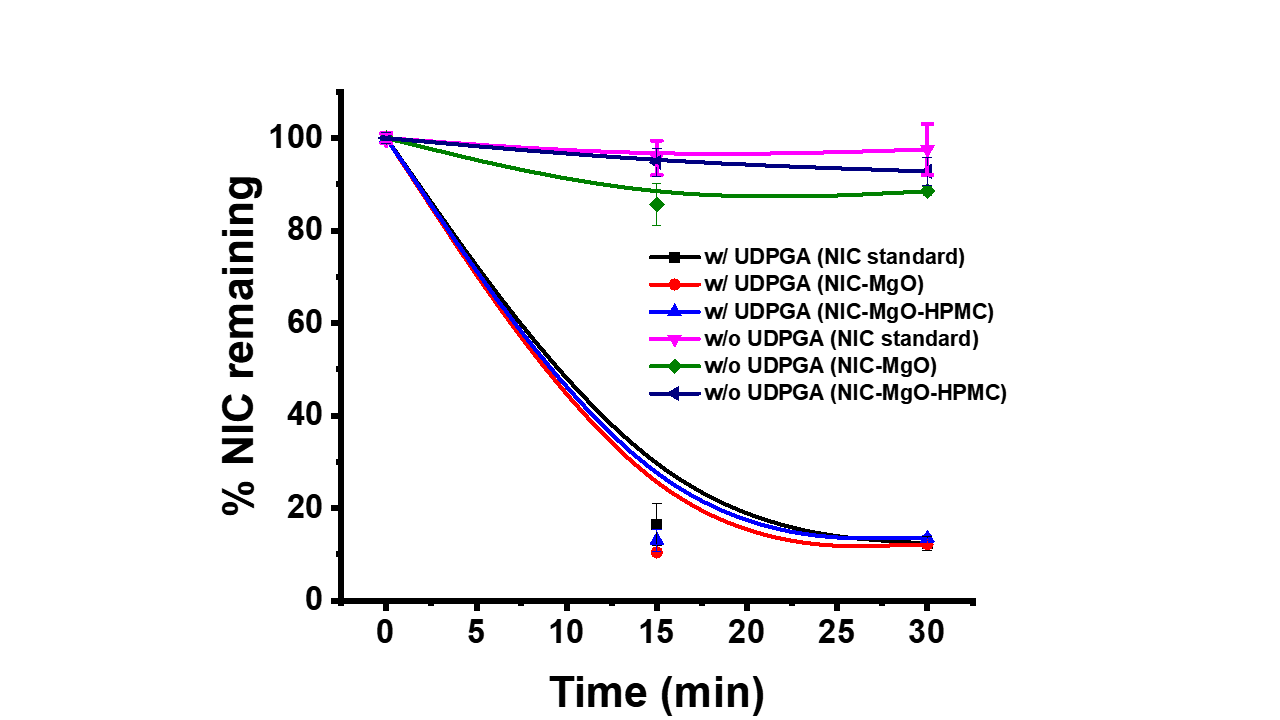
**

**Figure S1.** In vitro metabolic stability of niclosamide (NIC) standard, NIC-MgO, and NIC-MgO- hydroxypropyl methylcellulose (HPMC) in rat intestinal microsomes with or without UDP-glucuronic acid (UDPGA) treatment. (Reused with permission from Small, 2024)
